# Prostein expression in human tumors: a tissue microarray study on 19,202 tumors from 152 different Tumor entities

**DOI:** 10.1186/s13000-023-01434-5

**Published:** 2024-01-13

**Authors:** Florian Viehweger, Carola Böcker, Sören Weidemann, Morton Freytag, Anne Menz, Franziska Büscheck, Andreas M. Luebke, Devita Putri, Martina Kluth, Claudia Hube-Magg, Andrea Hinsch, Maximilian Lennartz, Florian Lutz, Viktor Reiswich, Doris Höflmayer, Christoph Fraune, Katharina Möller, Christian Bernreuther, Patrick Lebok, Guido Sauter, Stefan Steurer, David Dum, Andreas H. Marx, Ronald Simon, Till Krech, Till S. Clauditz, Frank Jacobsen, Natalia Gorbokon, Eike Burandt, Sarah Minner, Simon Kind

**Affiliations:** 1 Institute of Pathology, University Medical Center Hamburg-Eppendorf, Martinistr. 52, 20246 Hamburg, Germany; 2 Institute of Pathology, Clinical Center Osnabrueck, Am Finkenhuegel 1, Osnabrueck, 49076 Germany; 3 Department of Pathology, Academic Hospital Fuerth, Jakob-Henle-Straße 1, Fuerth, 90766 Germany

**Keywords:** Prostein, Tissue microarray, Immunohistochemistry, Human cancers

## Abstract

**Background:**

Prostein (P501S), also termed solute carrier family 45 member 3 (SLC45A3) is an androgen regulated protein which is preferentially expressed in prostate epithelial cells. Because of its frequent expression in prostate cancer, prostein was suggested a diagnostic prostate cancer marker.

**Methods:**

In order to comprehensively assess the diagnostic utility of prostein immunohistochemistry, a tissue microarray containing 19,202 samples from 152 different tumor types and subtypes as well as 608 samples of 76 different normal tissue types was analyzed by immunohistochemistry.

**Results:**

Prostein immunostaining was typically cytoplasmic, granular and perinuclear. Prostein positivity was seen in 96.7% of 419 prostate cancers including 78.3% with strong staining. In 16,709 extra-prostatic tumors, prostein positivity was observed in 7.2% of all cases but only 0.3% had a strong staining. Overall, 50 different extra-prostatic tumor categories were prostein positive, 12 of which included at least one strongly positive case. Extra-prostatic tumors with highest rates of prostein positivity included different subtypes of salivary gland tumors (7.6-44.4%), neuroendocrine neoplasms (15.8-44.4%), adenocarcinomas of the gastrointestinal tract (7.3-14.8%), biliopancreatic adenocarcinomas (3.6-38.7%), hepatocellular carcinomas (8.1%), and adenocarcinomas of other organs (up to 21%).

**Conclusions:**

Our data provide a comprehensive overview on prostein expression in human cancers. Prostein is a highly sensitive prostate cancer marker occurring in > 96% of prostate cancers. Because prostein can also be expressed in various other tumor entities, classifying of a tumor mass as a prostate cancer should not be based on prostein positivity alone.

**Supplementary Information:**

The online version contains supplementary material available at 10.1186/s13000-023-01434-5.

## Background

Prostein (P501S), also termed solute carrier family 45 member 3 (SLC45A3) is a protein composed of 553 amino acids which is coded by the SLC45A3 gene at chromosome 1q32.1 [1]. Its function is not well known but some data suggest a role in transmembrane transport of sugars [2]. Prostein is predominantly expressed in the prostate, where its expression is androgen regulated [3]. Prostein is the second most common 5′ partner gene in ETS Transcription Factor ERG (ERG) rearrangements in prostate cancer after Transmembrane Serine Protease 2 (TMPRSS2) [4, 5], another constitutively expressed androgen regulated gene in prostate epithelium [6]. In the brain, prostein plays a role in regulating the lipid metabolism of oligodendrocytes and myelin [7].

A high level of prostein expression is a common feature in prostate cancer. Amanda et al. [8] described prostein positivity in 97% of 59 analyzed prostate cancers. Queisser et al. [9] found prostein expression in 96% of 79 prostate cancers. Sheridan et al. [10] reported prostein positivity in 99% of 53 metastatic prostatic carcinomas. Based on these data, prostein immunohistochemistry (IHC) has been suggested as a diagnostic tool for the distinction of prostatic adenocarcinoma from other tumors. This notion is also supported by data describing high specificity of prostein expression for prostate cancer. For example, Garudadri et al. [11] described a 100% specificity of prostein IHC in a study on 100 prostatic carcinomas and 60 normal and cancerous extra-prostatic tissues. In an analysis of 600 tumors from 20 sites of origin, Mochizuki et al. [12] found prostein positivity in 30 of 30 prostate adenocarcinomas but in only one tumor each of 30 hepatocellular carcinomas and of 30 invasive breast cancers of no special type (NST). Kalos et al. [3] did not detect prostein staining in 3,454 samples of more than 130 tumor entities and subentities while 94% of 60 analyzed prostate cancers showed prostein positivity. Osunkoya et al. [13] did not find prostein positivity in any of 9 colorectal adenocarcinomas infiltrating the prostate. Srinivasan et al. [14] did not see any prostein positivity in 132 urothelial carcinomas. However, Arnesen et al. [15] found prostein positivity in 11 of 14 Sertoli-Leydig or Leydig cell tumors of the testis and ovary and Chuang et al. [16] reported prostein positivity in 7 of 41 invasive urothelial carcinomas.

To further corroborate the potential diagnostic utility of prostein IHC, a comprehensive survey of prostein immunostaining in an even broader range of tumor types is desirable. We therefore evaluated prostein expression in more than 19,000 tumor tissue samples from 152 different tumor types and subtypes as well as 76 different non-neoplastic tissue types by IHC in a tissue microarray (TMA) format.

## Materials and methods

### Tissue microarrays (TMAs)

Our normal tissue TMA was composed of 8 samples from 8 different donors for each of 76 different normal tissue types (608 samples on one slide). The cancer TMAs contained a total of 19,202 primary tumors from 152 tumor types and subtypes. The composition of both normal and cancer TMAs is described in detail in the “Results” section. Clinico-pathological data including pathological tumor stage (pT), grade, lymph node status (pN), lymphatic vessel (L) and blood vessel (V) infiltration were available for 327 gastric, 2,139 breast, and 2,351 colorectal carcinomas. All samples were from the archives of the Institutes of Pathology, University Hospital of Hamburg, Germany, the Institute of Pathology, Clinical Center Osnabrueck, Germany, and Department of Pathology, Academic Hospital Fuerth, Germany. Tissues were fixed in 4% buffered formalin and then embedded in paraffin. TMA tissue spot diameter was 0.6 mm. The use of archived remnants of diagnostic tissues for manufacturing of TMAs and their analysis for research purposes as well as patient data analysis has been approved by local laws (HmbKHG, § 12) and by the local ethics committee (Ethics commission Hamburg, WF-049/09). All work has been carried out in compliance with the Helsinki Declaration.

### Immunohistochemistry

Freshly cut TMA sections were immunostained on one day and in one experiment. Slides were deparaffinized with xylol, rehydrated through a graded alcohol series and exposed to heat-induced antigen retrieval for 5 min in an autoclave at 121 °C in pH 9.0 DakoTarget Retrieval Solution™ (Agilent, CA, USA; #S2367). Endogenous peroxidase activity was blocked with Dako Peroxidase Blocking Solution™ (Agilent, CA, USA; #52,023) for 10 min. Primary antibody specific for prostein (rabbit recombinant monoclonal, MSVA-460R, MS Validated Antibodies, Hamburg, Germany; #5241-460R) was applied at 37 °C for 60 min at a dilution of 1:150. For the purpose of antibody validation, the normal tissue TMA was also analyzed by the rabbit recombinant monoclonal prostein antibody EPR4795(2) (Abcam, Cambridge, UK; #ab137065) at a dilution of 1:150 and an otherwise identical protocol. Bound antibody was then visualized using the EnVision Kit™ (Agilent, CA, USA; #K5007) according to the manufacturer’s directions. The sections were counterstained with haemalaun. For normal tissues, the staining intensity of positive cells was semi-quantitively recorded (+, ++, +++). For tumor tissues, the percentage of prostein positive neoplastic cells was estimated, and the staining intensity was semi-quantitatively recorded (0, 1+, 2+, 3+). For statistical analyses, the staining results were categorized into four groups. Tumors without any staining were considered negative. Tumors with 1 + staining intensity in ≤ 70% of tumor cells or 2 + intensity in ≤ 30% of tumor cells were considered weakly positive. Tumors with 1 + staining intensity in > 70% of tumor cells, 2 + intensity in 31-70%, or 3 + intensity in ≤ 30% of tumor cells were considered moderately positive. Tumors with 2 + intensity in > 70% or 3 + intensity in > 30% of tumor cells werde considered strongly positive.

### Statistics

Statistical calculations were performed with JMP 16 software (SAS Institute Inc., NC, USA). Contingency tables and the chi²-test were performed to search for associations between prostein immunostaining and tumor phenotype.

## Results

### Technical issues

A total of 17,146 (89.3%) of 19,202 tumor samples were interpretable in our TMA analysis. Non-interpretable samples demonstrated lack of unequivocal tumor cells or loss of the tissue spot during technical procedures. A sufficient number of samples (≥ 4) of each normal tissue type was evaluable.

### Prostein in normal tissues

**Fig. 1:**
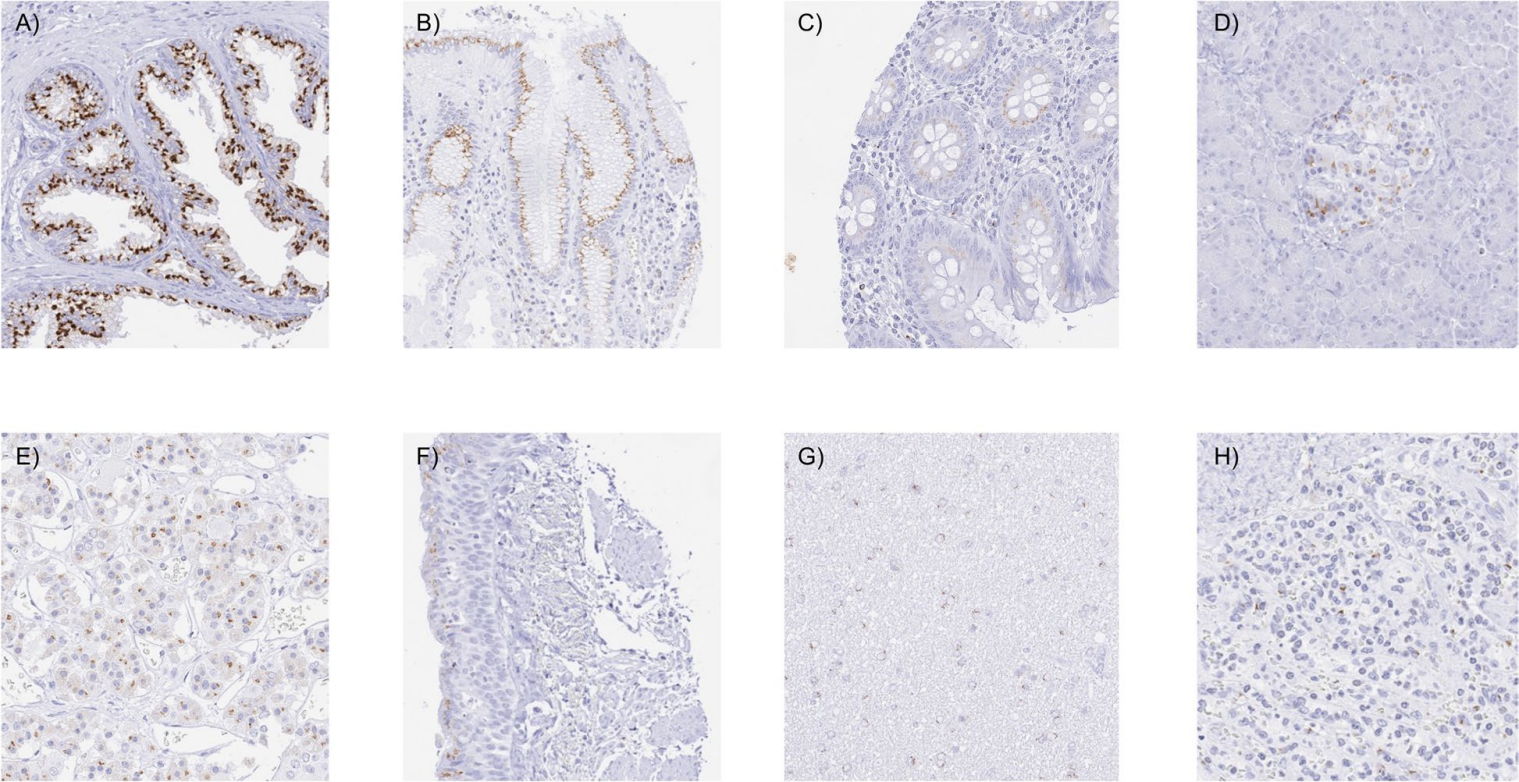
Prostein immunostaining of normal tissues. Prostein staining was always granular, cytoplasmic and predominantly perinuclear (“endoplasmatic reticulum pattern”). The panels show a particularly strong prostein staining of acinar cells of the prostate (**A**) while the staining is less intense in surface epithelium of the stomach (**B**). An even weaker prostein positivity (not always involving all samples and all cells) can also be seen in colorectal epithelium (**C**), pancreatic islet cells (**D**), epithelial cells of the adenohypophysis (**E**), respiratory epithelium of the lung (**F**), and in glia cells of the brain (**G**). An intense perinuclear prostein staining also occurs in a subset of monocytic cells of the spleen (**H**)

Prostein staining was always granular, cytoplasmic and predominantly perinuclear (“endoplasmatic reticulum pattern”). The staining was particularly strong in acinar cells of the prostate and occurred at lesser intensity in surface epithelial cells of the stomach, in goblet cells of the respiratory epithelium of the lung and (weaker) in bronchial glands, as well as in a subset of epithelial cells of the adenohypophysis. A weak prostein staining was also seen in few colorectal epithelial cells (not in all samples) and in a subset of pancreatic islet cells. A perinuclear granular cytoplasmic prostein positivity also occurred in a small fraction of (monocytic) cells in the spleen and in few cells of lymph nodes. In the brain, some glia cells showed a perinuclear granular cytoplasmic prostein staining. Representative images are shown in Fig. 1. All these findings were seen by both antibodies, MSVA-460R and EPR4795(2). An additional cytoplasmic staining in the placenta and in testicular cells of the spermatogenesis was only seen by EPR4795(2) (Supplementary Fig. 1) and therefore considered an antibody-specific cross-reactivity of EPR4795(2). Prostein immunostaning was absent in skeletal muscle, heart muscle, smooth muscle, myometrium of the uterus, corpus spongiosum of the penis, ovarian stroma, fat, skin (including hair follicles and sebaceous glands), oral mucosa of the lip, surface epithelium of the oral cavity and the tonsil, transitional mucosa of the anal canal, ectocervix, squamous epithelium of the esophagus, urothelium of the renal pelvis and urinary bladder, decidua, placenta, thymus, tonsil, gall bladder, liver, parotid gland, submandibular gland, sublingual gland, duodenum, small intestine, appendix, colorectum, kidney, seminal vesicle, testis, epididymis, breast, endocervix, endometrium, fallopian tube, adrenal gland, parathyroid gland, and the neurohypophysis.

### Prostein in cancer tissues

**Fig. 2:**
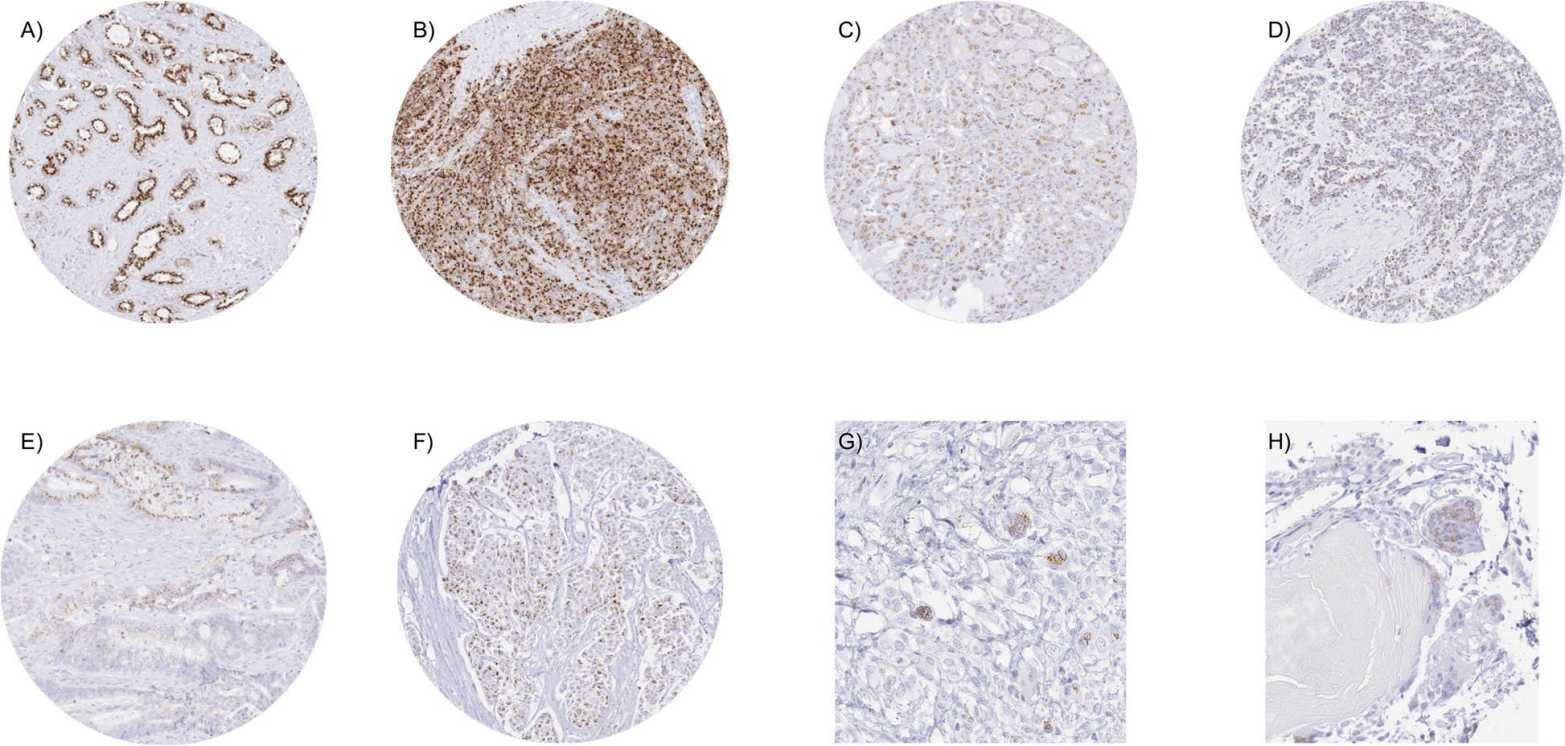
Prostein immunostaining in cancer. Prostein staining is usually granular, cytoplasmic and predominantly perinuclear (“endoplasmatic reticulum pattern”). The panels show a particularly strong prostein positivity in a Gleason 3 + 3 = 6 carcinoma (**A**) and a recurrent Gleason 5 + 5 = 10 carcinoma of the prostate (**B**). Prostein staining of tumor cells is less intense but still significant in samples of mucoepidermoid carcinoma of a salivary gland (**C**), neuroendocrine tumor of the lung (**D**), adenocarcinoma of the colon (**E**), and a muscle-invasive urothelial carcinoma of the urinary bladder (**F**). A distinct staining of giant cells is seen in samples of a giant cell tumor of the tendon sheet (**G**) and a pilomatrixoma of the skin (**H**)

**Fig. 3:**
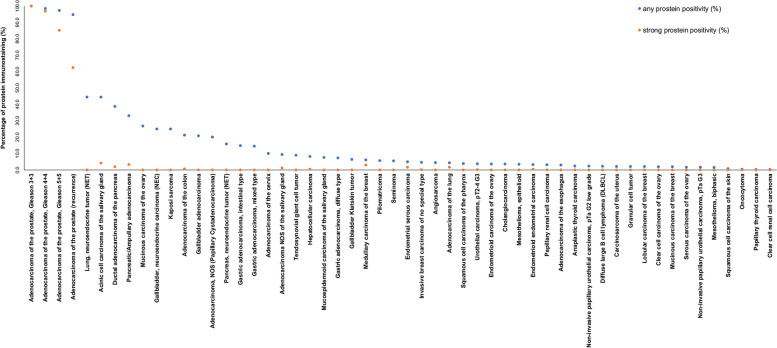
Ranking order of prostein immunostaining in tumors. Both the percentage of positive cases (blue dots) and the percentage of strongly positive cases (orange dots) are shown

Similarly, as in normal tissues, prostein immunostaining was typically cytoplasmic, granular and perinuclear in tumors. Prostein positivity, and especially a strong prostein staining was predominantly seen in prostatic adenocarcinomas. 93% of primary prostate cancers and 63% of recurrent prostate cancers showed a strong prostein immunostaining while 98% of primary prostate cancers and 94% of recurrent prostate cancers showed at least a weak positivity. Prostein staining was absent in all 18 small cell neuroendocrine carcinomas of the prostate. Prostein positivity - mostly at a lower level - was also detectable in 1,204 (7.2%) of the 16,709 analyzable extra-prostatic tumors. Of these, 922 (5.5%) showed a weak, 239 (1.4%) a moderate, and only 43 (0.3%) a strong immunostaining. Overall, 50 (34.0%) of 157 extra-prostatic tumor categories showed detectable prostein expression with 12 (8.2%) tumor categories including at least one strongly positive tumor (Table 1). Representative images of prostein positive tumors are shown in Fig. 2. Extra-prostatic tumors with highest rate of prostein positivity included different subtypes of salivary gland tumors (7.6-44.4%), neuroendocrine neoplasms (15.8-44.4%), adenocarcinomas of the gastrointestinal tract (7.3-14.8%), and biliopancreatic adenocarcinomas (3.6-38.7%), hepatocellular carcinomas (8.1%), as well as adenocarcinomas of other organs of origin (up to 21%). A graphical representation of a ranking order of prostein positive and strongly positive cancers is given in Fig. 3. A comparison between prostein expression and tumor phenotype is shown in Table 2. Detectable prostein expression was linked to high grade (*p* = 0.0105), HER2 positivity (*p* = 0.0312), and estrogen receptor negativity (*p* = 0.0330) in invasive breast carcinomas of no special type (NST), V0 status (*p* = 0.0139), right sided tumor location (*p* = 0.0479), and KRAS mutations (*p* = 0.0133) in colorectal cancer, pN0 stage (*p* = 0.0424) in pancreatic ductal adenocarcinoma as well as to microsatellite instability in gastric cancers (*p* = 0.0015).

**Table 1:** Prostein immunostaining in human tumors

			Prostein immunostaining result				
	Tumor entity	on TMA (n)	analyzable (n)	negative (%)	weak (%)	moderate (%)	strong (%)
Tumors of the skin	Pilomatricoma	35	35	94.3	2.9	2.9	0.0
	Basal cell carcinoma	89	58	100.0	0.0	0.0	0.0
	Benign nevus	29	25	100.0	0.0	0.0	0.0
	Squamous cell carcinoma of the skin	145	129	99.2	0.8	0.0	0.0
	Malignant melanoma	65	61	100.0	0.0	0.0	0.0
	Malignant melanoma lymph node metastasis	86	73	100.0	0.0	0.0	0.0
	Merkel cell carcinoma	48	48	100.0	0.0	0.0	0.0
Tumors of the head and neck	Squamous cell carcinoma of the larynx	109	96	100.0	0.0	0.0	0.0
	Squamous cell carcinoma of the pharynx	60	51	96.1	3.9	0.0	0.0
	Oral squamous cell carcinoma (floor of the mouth)	130	115	100.0	0.0	0.0	0.0
	Pleomorphic adenoma of the parotid gland	50	48	100.0	0.0	0.0	0.0
	Warthin tumor of the parotid gland	104	100	100.0	0.0	0.0	0.0
	Adenocarcinoma, NOS (Papillary Cystadenocarcinoma)	14	10	80.0	10.0	10.0	0.0
	Salivary duct carcinoma	15	12	100.0	0.0	0.0	0.0
	Acinic cell carcinoma of the salivary gland	181	144	55.6	22.2	18.1	4.2
	Adenocarcinoma NOS of the salivary gland	109	85	90.6	3.5	4.7	1.2
	Adenoid cystic carcinoma of the salivary gland	180	113	100.0	0.0	0.0	0.0
	Basal cell adenocarcinoma of the salivary gland	25	23	100.0	0.0	0.0	0.0
	Basal cell adenoma of the salivary gland	101	85	100.0	0.0	0.0	0.0
	Epithelial-myoepithelial carcinoma of the salivary gland	53	51	100.0	0.0	0.0	0.0
	Mucoepidermoid carcinoma of the salivary gland	343	291	92.4	3.4	4.1	0.0
	Myoepithelial carcinoma of the salivary gland	21	18	100.0	0.0	0.0	0.0
	Myoepithelioma of the salivary gland	11	9	100.0	0.0	0.0	0.0
	Oncocytic carcinoma of the salivary gland	12	12	100.0	0.0	0.0	0.0
	Polymorphous adenocarcinoma, low grade, of the salivary gland	41	27	100.0	0.0	0.0	0.0
	Pleomorphic adenoma of the salivary gland	53	40	100.0	0.0	0.0	0.0
Tumors of the lung, pleura and thymus	Adenocarcinoma of the lung	196	187	95.7	2.1	0.5	1.6
	Squamous cell carcinoma of the lung	80	71	100.0	0.0	0.0	0.0
	Small cell carcinoma of the lung	16	16	100.0	0.0	0.0	0.0
	Mesothelioma, epithelioid	40	29	96.6	3.4	0.0	0.0
	Mesothelioma, biphasic	77	71	98.6	1.4	0.0	0.0
	Thymoma	29	28	100.0	0.0	0.0	0.0
	Lung, neuroendocrine tumor (NET)	29	27	55.6	14.8	29.6	0.0
Tumors of the female genital tract	Squamous cell carcinoma of the vagina	78	65	100.0	0.0	0.0	0.0
	Squamous cell carcinoma of the vulva	157	141	100.0	0.0	0.0	0.0
	Squamous cell carcinoma of the cervix	136	126	100.0	0.0	0.0	0.0
	Adenocarcinoma of the cervix	23	20	90.0	10.0	0.0	0.0
	Endometrioid endometrial carcinoma	338	272	96.7	2.6	0.4	0.4
	Endometrial serous carcinoma	86	62	95.2	3.2	0.0	1.6
	Carcinosarcoma of the uterus	57	47	97.9	2.1	0.0	0.0
	Endometrial carcinoma, high grade, G3	13	10	100.0	0.0	0.0	0.0
	Endometrial clear cell carcinoma	9	5	100.0	0.0	0.0	0.0
	Endometrioid carcinoma of the ovary	130	111	96.4	3.6	0.0	0.0
	Serous carcinoma of the ovary	580	540	98.3	1.5	0.2	0.0
	Mucinous carcinoma of the ovary	101	86	73.3	12.8	14.0	0.0
	Clear cell carcinoma of the ovary	51	51	98.0	2.0	0.0	0.0
	Carcinosarcoma of the ovary	47	46	100.0	0.0	0.0	0.0
	Granulosa cell tumor of the ovary	44	38	100.0	0.0	0.0	0.0
	Leydig cell tumor of the ovary	4	4	100.0	0.0	0.0	0.0
	Sertoli cell tumor of the ovary	1	1	100.0	0.0	0.0	0.0
	Sertoli Leydig cell tumor of the ovary	3	3	100.0	0.0	0.0	0.0
	Steroid cell tumor of the ovary	3	3	100.0	0.0	0.0	0.0
	Brenner tumor	41	41	100.0	0.0	0.0	0.0
Tumors of the breast	Invasive breast carcinoma of no special type	1764	1656	95.5	3.8	0.7	0.1
	Lobular carcinoma of the breast	363	336	97.9	2.1	0.0	0.0
	Medullary carcinoma of the breast	34	33	93.9	3.0	0.0	3.0
	Tubular carcinoma of the breast	29	25	100.0	0.0	0.0	0.0
	Mucinous carcinoma of the breast	65	52	98.1	1.9	0.0	0.0
	Phyllodes tumor of the breast	50	40	100.0	0.0	0.0	0.0
Tumors of the digestive system	Adenomatous polyp, low-grade dysplasia	50	50	100.0	0.0	0.0	0.0
	Adenomatous polyp, high-grade dysplasia	50	50	100.0	0.0	0.0	0.0
	Adenocarcinoma of the colon	2483	2220	78.8	17.7	2.9	0.5
	Gastric adenocarcinoma, diffuse type	215	192	92.7	6.8	0.5	0.0
	Gastric adenocarcinoma, intestinal type	215	203	85.2	10.3	4.4	0.0
	Gastric adenocarcinoma, mixed type	62	62	85.5	12.9	1.6	0.0
	Adenocarcinoma of the esophagus	83	66	97.0	3.0	0.0	0.0
	Squamous cell carcinoma of the esophagus	76	59	100.0	0.0	0.0	0.0
	Squamous cell carcinoma of the anal canal	91	80	100.0	0.0	0.0	0.0
	Cholangiocarcinoma	58	56	96.4	3.6	0.0	0.0
	Gallbladder adenocarcinoma	51	48	79.2	12.5	8.3	0.0
	Gallbladder Klatskin tumor	42	31	93.5	6.5	0.0	0.0
	Hepatocellular carcinoma	312	270	91.9	6.3	1.5	0.4
	Ductal adenocarcinoma of the pancreas	659	625	61.3	28.8	8.0	1.9
	Pancreatic/Ampullary adenocarcinoma	98	94	67.0	24.5	5.3	3.2
	Acinar cell carcinoma of the pancreas	18	18	100.0	0.0	0.0	0.0
	Gastrointestinal stromal tumor (GIST)	62	61	100.0	0.0	0.0	0.0
	Appendix, neuroendocrine tumor (NET)	25	20	0.0	0.0	0.0	0.0
	Colorectal, neuroendocrine tumor (NET)	12	11	100.0	0.0	0.0	0.0
	Ileum, neuroendocrine tumor (NET)	53	53	100.0	0.0	0.0	0.0
	Pancreas, neuroendocrine tumor (NET)	101	95	84.2	6.3	9.5	0.0
	Colorectal, neuroendocrine carcinoma (NEC)	14	12	100.0	0.0	0.0	0.0
	Ileum, neuroendocrine carcinoma (NEC)	8	8	100.0	0.0	0.0	0.0
	Gallbladder, neuroendocrine carcinoma (NEC)	4	4	75.0	0.0	25.0	0.0
	Pancreas, neuroendocrine carcinoma (NEC)	14	14	100.0	0.0	0.0	0.0
Tumors of the urinary system	Non-invasive papillary urothelial carcinoma, pTa G2 low grade	177	172	97.7	1.7	0.6	0.0
	Non-invasive papillary urothelial carcinoma, pTa G2 high grade	141	139	100.0	0.0	0.0	0.0
	Non-invasive papillary urothelial carcinoma, pTa G3	219	128	98.4	1.6	0.0	0.0
	Urothelial carcinoma, pT2-4 G3	735	630	96.3	3.0	0.5	0.2
	Squamous cell carcinoma of the bladder	22	18	100.0	0.0	0.0	0.0
	Small cell neuroendocrine carcinoma of the bladder	23	23	100.0	0.0	0.0	0.0
	Sarcomatoid urothelial carcinoma	25	19	100.0	0.0	0.0	0.0
	Urothelial carcinoma of the kidney pelvis	62	54	100.0	0.0	0.0	0.0
	Clear cell renal cell carcinoma	1287	1135	99.7	0.3	0.0	0.0
	Papillary renal cell carcinoma	368	325	96.9	2.2	0.9	0.0
	Clear cell (tubulo) papillary renal cell carcinoma	26	24	100.0	0.0	0.0	0.0
	Chromophobe renal cell carcinoma	170	149	100.0	0.0	0.0	0.0
	Oncocytoma	257	228	99.6	0.4	0.0	0.0
Tumors of the male genital organs	Adenocarcinoma of the prostate, Gleason 3 + 3	83	74	0.0	0.0	0.0	100.0
	Adenocarcinoma of the prostate, Gleason 4 + 4	80	64	1.6	1.6	0.0	96.9
	Adenocarcinoma of the prostate, Gleason 5 + 5	85	74	2.7	2.7	9.5	85.1
	Adenocarcinoma of the prostate (recurrence)	258	207	5.3	17.4	15.0	62.3
	Small cell neuroendocrine carcinoma of the prostate	19	18	100.0	0.0	0.0	0.0
	Seminoma	682	673	94.5	5.1	0.4	0.0
	Embryonal carcinoma of the testis	54	49	100.0	0.0	0.0	0.0
	Leydig cell tumor of the testis	31	23	100.0	0.0	0.0	0.0
	Sertoli cell tumor of the testis	2	1	100.0	0.0	0.0	0.0
	Sex cord stromal tumor of the testis	1	1	100.0	0.0	0.0	0.0
	Spermatocytic tumor of the testis	1	1	100.0	0.0	0.0	0.0
	Yolk sac tumor	53	45	100.0	0.0	0.0	0.0
	Teratoma	53	45	100.0	0.0	0.0	0.0
	Squamous cell carcinoma of the penis	92	71	100.0	0.0	0.0	0.0
Tumors of endocrine organs	Adenoma of the thyroid gland	113	110	100.0	0.0	0.0	0.0
	Papillary thyroid carcinoma	391	354	99.7	0.3	0.0	0.0
	Follicular thyroid carcinoma	154	146	100.0	0.0	0.0	0.0
	Medullary thyroid carcinoma	111	105	100.0	0.0	0.0	0.0
	Parathyroid gland adenoma	43	32	100.0	0.0	0.0	0.0
	Anaplastic thyroid carcinoma	45	42	97.6	2.4	0.0	0.0
	Adrenal cortical adenoma	50	48	100.0	0.0	0.0	0.0
	Adrenal cortical carcinoma	28	28	100.0	0.0	0.0	0.0
	Phaeochromocytoma	50	50	100.0	0.0	0.0	0.0
Tumors of haemotopoetic and lymphoid tissues	Hodgkin Lymphoma	103	94	100.0	0.0	0.0	0.0
	Small lymphocytic lymphoma, B-cell type (B-SLL/B-CLL)	50	39	100.0	0.0	0.0	0.0
	Diffuse large B cell lymphoma (DLBCL)	113	92	97.8	2.2	0.0	0.0
	Follicular lymphoma	88	65	100.0	0.0	0.0	0.0
	T-cell Non Hodgkin lymphoma	25	20	100.0	0.0	0.0	0.0
	Mantle cell lymphoma	18	12	100.0	0.0	0.0	0.0
	Marginal zone lymphoma	16	12	100.0	0.0	0.0	0.0
	Diffuse large B-cell lymphoma (DLBCL) in the testis	16	15	100.0	0.0	0.0	0.0
	Burkitt lymphoma	5	1	100.0	0.0	0.0	0.0
Tumors of soft tissue and bone	Tendosynovial giant cell tumor	45	45	91.1	8.9	0.0	0.0
	Granular cell tumor	53	47	97.9	2.1	0.0	0.0
	Leiomyoma	50	50	100.0	0.0	0.0	0.0
	Leiomyosarcoma	94	90	100.0	0.0	0.0	0.0
	Liposarcoma	145	144	100.0	0.0	0.0	0.0
	Malignant peripheral nerve sheath tumor (MPNST)	15	14	100.0	0.0	0.0	0.0
	Myofibrosarcoma	26	26	100.0	0.0	0.0	0.0
	Angiosarcoma	74	67	95.5	1.5	3.0	0.0
	Angiomyolipoma	91	89	100.0	0.0	0.0	0.0
	Dermatofibrosarcoma protuberans	21	16	100.0	0.0	0.0	0.0
	Ganglioneuroma	14	14	100.0	0.0	0.0	0.0
	Kaposi sarcoma	8	4	75.0	25.0	0.0	0.0
	Neurofibroma	117	117	100.0	0.0	0.0	0.0
	Sarcoma, not otherwise specified (NOS)	74	68	100.0	0.0	0.0	0.0
	Paraganglioma	41	41	100.0	0.0	0.0	0.0
	Ewing sarcoma	23	16	100.0	0.0	0.0	0.0
	Rhabdomyosarcoma	7	6	100.0	0.0	0.0	0.0
	Schwannoma	122	121	100.0	0.0	0.0	0.0
	Synovial sarcoma	12	11	100.0	0.0	0.0	0.0
	Osteosarcoma	44	41	100.0	0.0	0.0	0.0
	Chondrosarcoma	40	38	100.0	0.0	0.0	0.0
	Rhabdoid tumor	5	5	100.0	0.0	0.0	0.0
	Solitary fibrous tumor	17	17	100.0	0.0	0.0	0.0

**Table 2:** Prostein and tumor phenotype

			Prostein immunostaining result				
		n	negative (%)	weak (%)	moderate (%)	strong (%)	*P*
Invasive breast carcinoma of no special type	pT1	774	95.9	3.5	0.6	0	0.2176
	pT2	626	94.9	4.3	0.8	0	
	pT3-4	125	93.6	5.6	0	0.8	
	G1	191	96.3	3.1	0.5	0	0.0105
	G2	817	96.9	2.7	0.4	0	
	G3	562	92.3	6.2	1.2	0.2	
	pN0	698	95.3	4.4	0.3	0	0.1691
	pN+	527	94.7	4.2	1	0.2	
	pM0	198	96.5	3	0.5	0	0.5637
	pM1	116	94.8	3.4	1.7	0	
	HER2 negative	889	96.4	3	0.6	0	0.0312
	HER2 positive	124	91.9	4.8	3.2	0	
	ER negative	215	92.1	6	1.9	0	0.033
	ER positive	746	96.5	2.8	0.7	0	
	PR negative	414	94.2	4.6	1.2	0	0.1836
	PR positive	594	96.6	2.7	0.7	0	
	non-triple negative	786	95.8	3.2	1	0	0.5848
	triple negative	144	94.4	4.9	0.7	0	
Adenocarcinoma of the pancreas	pT1	16	75	18.8	6.3	0	0.7582
	pT2	71	60.6	25.4	11.3	2.8	
	pT3	384	60.9	29.7	7.3	2.1	
	pT4	30	70	16.7	10	3.3	
	G1	17	52.9	35.3	11.8	0	0.7482
	G2	353	61.8	28	7.4	2.8	
	G3	108	62	30.6	6.5	0.9	
	pN0	108	58.3	24.1	14.8	2.8	0.0424
	pN+	392	62.5	29.3	6.1	2	
	R0	253	62.1	26.1	9.1	2.8	0.5101
	R1	208	63	27.9	8.2	1	
	MMR proficient	453	61.8	28	7.7	2.4	0.8875
	MMR deficient	3	66.7	33.3	0	0	
Adenocarcinoma of the stomach	pT1-2	63	84.1	9.5	6.3	0	0.4894
	pT3	126	85.7	11.1	3.2	0	
	pT4	126	84.9	13.5	1.6	0	
	pN0	86	87.2	9.3	3.5	0	0.8345
	pN+	223	86.1	10.8	3.2	0.0	
	MMR proficient	40	70	15	15	0	0.0015
	MMR deficient	259	85.7	12.7	1.5	0	
Adenocarcinoma of the colon	pT1	80	78.8	18.8	1.3	1.3	0.0061
	pT2	414	70.5	25.6	3.1	0.7	
	pT3	1195	81.1	15.6	2.9	0.4	
	pT4	416	79.1	17.8	2.4	0.7	
	pN0	1101	77.7	17.9	3.7	0.6	0.0608
	pN+	993	79.5	18.2	1.8	0.5	
	V0	1514	77.8	18.1	3.4	0.7	0.0139
	V1	546	81.3	17.2	1.3	0.2	
	L0	684	80	15.8	3.7	0.6	0.1454
	L1	1387	78.1	19	2.4	0.6	
	right side	452	75.4	19.5	3.8	1.3	0.0479
	left side	1187	80.5	16.7	2.4	0.4	
	MMR proficient	1104	79.3	17.7	2.4	0.6	0.5061
	MMR deficient	85	77.6	17.6	4.7	0	
	RAS wildtype	422	85.5	12.8	1.4	0.2	0.0133
	RAS mutation	328	77.4	17.7	4	0.9	
	BRAF wildtype	123	79.7	16.3	1.6	2.4	0.6308
	BRAF V600E mutation	16	75	12.5	6.3	6.3	

*Abbreviation*: *pT* Pathological tumor stage, *G* Grade, *pN* Pathological lymph node status, *pM* Pathological status of distant metastasis, *R* Resection margin status, *V* Venous invasion, *L* Lymphatic invasion, *PR* Progesteron receptor, *MMR* Mismatch repair, *ER* Estrogen receptor

## Discussion

Our successful analysis of more than 17,000 tumors provided a comprehensive overview on the patterns of prostein expression in cancer. The predominant expression of prostein in prostate cancer was expected since studies analyzing 9-220 tumor cases had earlier identified prostein positivity in up to 100% of prostate cancers [4, 11, 17, 18]. Our positivity rate of 100% in Gleason 3 + 3 = 6, 98% in Gleason 4 + 4 = 8 and 97% in Gleason 5 + 5 = 10 prostate cancers is comparable with results from most previous studies [3, 19]. The concept that prostein IHC can be used to corroborate a suspected prostatic origin of a cancer tissue is further supported by the retained prostein expression in at least 80% of prostate cancers that recurred after hormonal therapy [19]. Sheridan et al. [10] had previously identified prostein positivity in 99% of 53 analyzed prostatic cancer metastases. Hernandez-Llodra et al. [4] have previously suggested that the few prostate cancers with reduced or absent prostein expression might harbor SLC45A3:ERG fusions and that these tumors may be characterized by poor prognosis.

The extensive analysis of non-prostatic tumors in this study identified a considerable number of tumor entities that can also express prostein. Although prostein expression was less frequent and often at markedly lower level in these tumors than in prostate cancer, the characteristic staining pattern with a distinct granular, perinuclear cytoplasmic prostein staining was always retained. The most commonly prostein positive tumors included salivary gland tumors, neuroendocrine neoplasms, various categories of gastrointestinal or biliopancreatic adenocarcinomas, hepatocellular carcinomas as well as adenocarcinomas of other organs of origin. All these tumor entities represent diagnostic options in case of a prostein positive tumor mass. It is of note that in some tumor entities, a perinuclear prostein expression was also observed in cells of monocytic origin such as for example in epitheloid cells accompanying lymphomas or in giant cells of tendon sheath tumors or in pilomatricoma. These findings fit with our observation of prostein positive monocytic cells in the spleen and the lymph node. Our data in primary and recurrent prostate cancer suggest sensitivity of 94–98% for the identification of a prostatic cancer origin, although these numbers might represent a slight underestimate because of an overrepresentation of Gleason 4 + 4, 5 + 5 and recurrent prostate cancers in our cohort. Accordingly, the sensitivity of PSAP (96.5%) and PSA (99.8%) were slightly higher in previous studies of our group analyzing large consecutive prostate cancer cohorts including much higher proportions of Gleason 3 + 3 and 3 + 4 cancer than in the current set of tumors. The specificity for the distinction of prostate cancer was somewhat lower for prostein (91.7%) as compared to the 100% for PSAP and PSA (99.7%) observed in these earlier studies [20, 21]. However, the characteristic granular perinuclear staining pattern that can hardly result from staining artefacts is a major strongpoint of prostein IHC which may thus justify the use of prostein antibodies as a part of a diagnostic panel for the identification of a prostatic cancer origin.

The location of the prostein protein in subcellular vesicles in the cytoplasm and co-localization to other compartments, i.e., the endoplasmatic reticulum fits well with the estimated function of prostein as a sucrose transport protein [2, 22]. However, many of the extra-prostatic tumor entities that were most commonly prostein positive were adenocarcinomas or neuroendocrine tumors. As these cell types share a secretory or neurosecretory function it might be speculated that prostein may have also a general role in cell secretion. The comparison of detectable prostein expression with histopathological and molecular tumor parameters in breast, colon, gastric and pancreatic adenocarcinoma had revealed only few statistically significant associations which do not provide strong evidence for a relevant biological/clinical role of prostein in non-prostatic cancers. It is possible that these findings represent statistical artifacts attributed to the high number of statistical analyses executed in this study.

Considering the large scale of our study, our assay was extensively validated by comparing our IHC findings in normal tissues with data obtained by another independent anti-prostein antibody and RNA data derived from three different publicly accessible databases [22–25]. To ensure an as broad as possible range of proteins to be tested for a possible cross-reactivity, 76 different normal tissues categories were included in this analysis. The validity of our assay was supported by the finding of the highest levels of prostein immunostaining in the prostate, the organ with the highest documented RNA expression level and the finding of prostein positive cell populations in most other organs with documented low level RNA expression such as the stomach, respiratory epithelium, hypophysis, spleen, and the brain. Only RNA expression in the liver could not be corroborated by our assay. That all prostein positive cell types detected by MSVA-460R (islet cells of the pancreas, respiratory epithelium, epithelial cells of the adenohypophysis, surface epithelial cells of the stomach, glia cells in the brain, monocytic cells in the spleen and lymph nodes) were also identified by the independent second antibody EPR4795(2) (Supplementary Fig. 1) adds further evidence for the validity of our assay. Additional stainings of the placenta and the testis which were only observed by EPR4795(2) were considered antibody specific cross-reactivities of this antibody and suggest that this antibody is less appropriate for prostein assessment.

## Conclusion

Our data provide a comprehensive overview on prostein expression in human cancers. The data show that prostein is a highly sensitive prostate cancer marker with positive results in at least 98% of primary prostate cancers. Because prostein can also be expressed in various other tumor entities, the classification of a tumor mass as a prostate cancer should not be made based on prostein positivity alone.

### Supplementary Information

**Additional file 1: Supplementary Fig. 1.** IHC validation by comparison of antibodies. The panels demonstrate a confirmation of all prostein stainings obtained by MSVA-460R by the independent antibody EPR4795(2). Using MSVA-460R, a granular, predominantly perinuclear staining was seen in epithelial cells of the prostate (A), stomach surface (B), respiratory epithelium (C), the adenohypophysis (D), and of pancreatic islets (E), as well as in some monocytic cells of the spleen (F) while staining was lacking in the first trimenon placenta (G) and the testis (H). Using clone EPR4795(2), identical cell types stained in the prostate (I), stomach (K), respiratory epithelium (L), adenohypophysis (M), pancreatic islets (N), and in the spleen (O). A cytoplasmic staining in the placenta (P) and in testicular cells of the spermatogenesis (Q) was only seen by EPR4795(2) and therefore considered an antibody-specific cross-reactivity of EPR4795(2). The images A-H and I-Q are from consecutive tissue sections.

## Data Availability

All data generated or analyzed during this study are included in this published article.

## Abbreviations

ERG: ETS Transcription Factor ERG
IHC: Immunohistochemistry
SCL45A3: Solute carrier family 45 member 3
TMA: Tissue microarray
TMPRSS2: Transmembrane Serine Protease 2
