# Supplementary figures and images for: Prostein expression in human tumors: a tissue microarray study on 19,202 tumors from 152 different Tumor entities
Source: Diagn Pathol. 2024 Jan 13;19:12. doi: 10.1186/s13000-023-01434-5 (PMC10788021; doi:10.1186/s13000-023-01434-5)

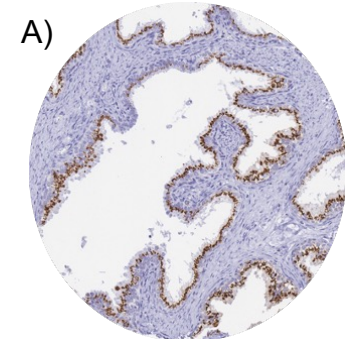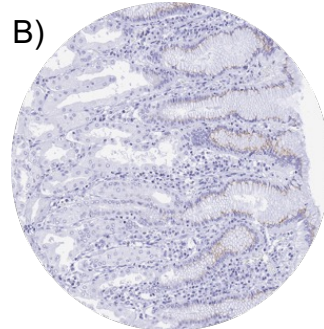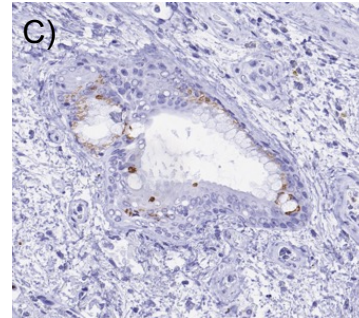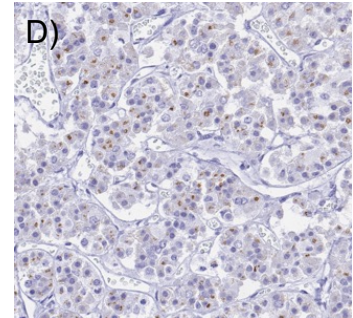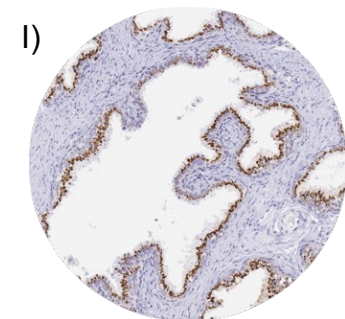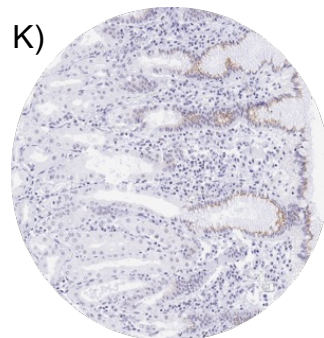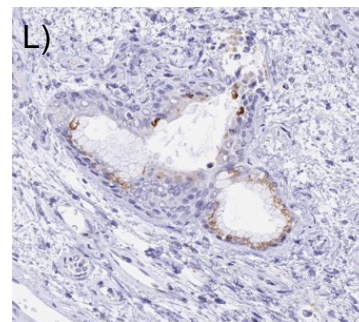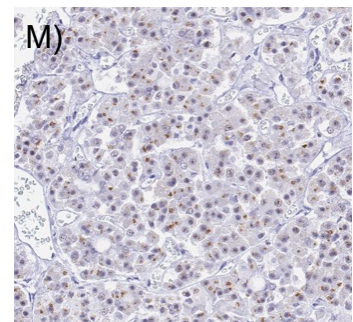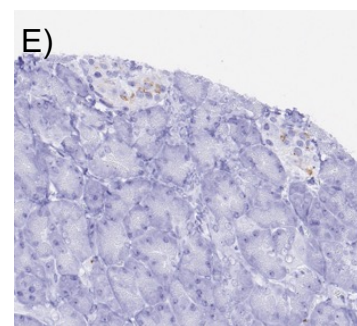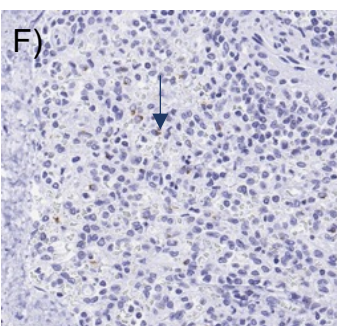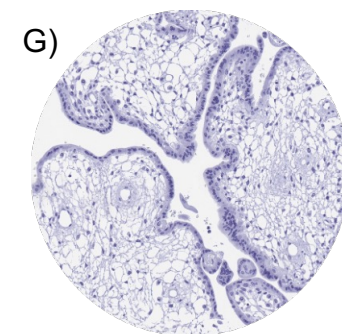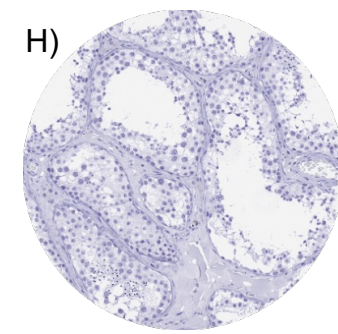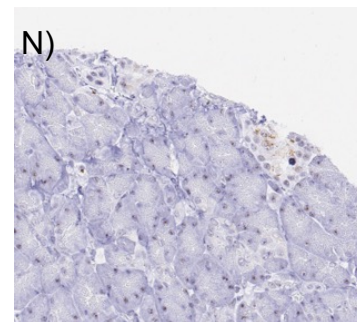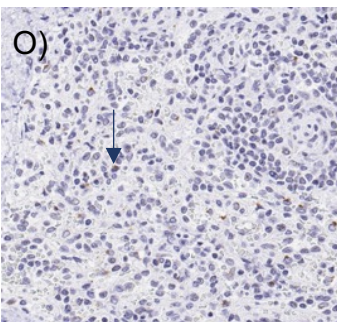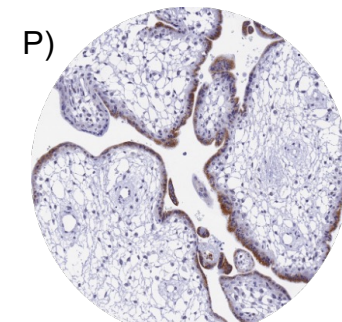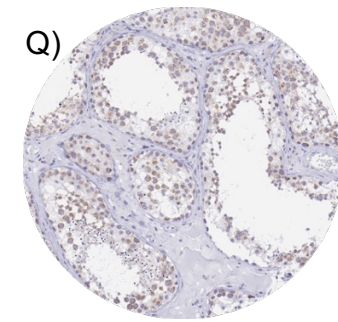

Supplement: Supplementary file 1 — Additional file 1: Supplementary Fig. 1. IHC validation by comparison of antibodies. The panels demonstrate a confirmation of all prostein stainings obtained by MSVA-460R by the independent antibody EPR4795(2). Using MSVA-460R, a granular, predominantly perinuclear staining was seen in epithelial cells of the prostate (A), stomach surface (B), respiratory epithelium (C), the adenohypophysis (D), and of pancreatic islets (E), as well as in some monocytic cells of the spleen (F) while staining was lacking in the first trimenon placenta (G) and the testis (H). Using clone EPR4795(2), identical cell types stained in the prostate (I), stomach (K), respiratory epithelium (L), adenohypophysis (M), pancreatic islets (N), and in the spleen (O). A cytoplasmic staining in the placenta (P) and in testicular cells of the spermatogenesis (Q) was only seen by EPR4795(2) and therefore considered an antibody-specific cross-reactivity of EPR4795(2). The images A-H and I-Q are from consecutive tissue sections. [file 13000_2023_1434_MOESM1_ESM.pdf]
